# Accelerating Nipah virus vaccine development to prevent future global outbreaks

**DOI:** 10.1097/MS9.0000000000005209

**Published:** 2026-06-03

**Authors:** Qadeer Ahmed, Shan e Ali Shoukat, Fatima Sohail, Muhammad Hanif Khan

**Affiliations:** a Department of Medicine, Karachi Medical and Dental College, Karachi, Pakistan; b Department of Medicine, Jinnah Sindh Medical University, Karachi, Pakistan; c Department of Medicine, Spinghar University, Kabul, Afghanistan

The Nipah virus (NiV) poses a serious threat to public health, particularly in South and Southeast Asia. NiV is a zoonotic paramyxovirus with a high case fatality rate (40–75%) and currently has no licensed vaccines or specific antiviral therapy^[^1^]^. Since its discovery in Malaysia in 1998, repeated outbreaks in Bangladesh and India have demonstrated the virus’s potential for both endemicity and epidemic spread^[^2,3^]^. Transmission occurs from fruit bats (*Pteropus* spp.), the natural reservoirs, to humans via contaminated food, direct contact with infected animals, or occasionally, human-to-human transmission^[^4,5^]^.

The clinical effect of NiV is dramatic and often manifests with neurological and respiratory disease. The disease usually manifests with fever, headache, and myalgia that can rapidly evolve to acute encephalitis, seizures, confusion, and coma^[^4^]^. Respiratory symptoms, such as coughing, shortness of breath, and acute respiratory distress, are frequent, especially in outbreaks that present with high mortality^[^2,4^]^. Survivors often experience long-term sequelae in the central nervous system, including cognitive deficits and motor abnormalities, emphasizing the morbidity of the virus^[^4^]^.

It is important to delineate the pathogenesis and transmission of NiV, as this could greatly aid in prevention and control. Viral entry is mediated by ephrin-B2 and -B3 receptor-dependent endothelial, epithelial, and neural cells, which can result in wide-ranging viral replication and inflammation^[^5^]^. As a result, environmental phenomena, such as deforestation, expansion of agriculture, and increased human-bat contact, can enhance the risk of spillover and recurrent losses in endemic areas^[^6^]^. Despite being less efficient compared to respiratory airborne viruses, human-to-human transmission has been reported within household and healthcare environments^[^3,5^]^.

Significant research gaps and preparedness deficits persist, with no available vaccines or specific antivirals and treatments, despite a defined epidemic potential^[^1^]^. A few vaccine candidates are being tested, but advances are impeded due to the unpredictable occurrence of infection and financial limitations^[^5,7^]^. A portfolio of important candidates is being utilized by the Coalition for Epidemic Preparedness Innovations (CEPI) to expedite the development of a Nipah vaccine. Phase II studies for the ChAdOx1 Nipah B candidate, which was created by the University of Oxford in partnership with the Serum Institute of India, have been funded with $7.3 million^[^8^]^. To facilitate quick outbreak deployment, a research reserve with up to 100 000 doses has been set up. Another contender, PHV02, created by Public Health Vaccines, has received $17.3 million in funding for its Phase II trials assessing safety and immunogenicity^[^9^]^. These programs, which have about 575 Bangladeshi participants, use COVID-proven systems to fill financing gaps and facilitate quick scale-up in high-risk locations. There are diagnostic techniques, but these may be unavailable in resource-limited settings, delaying early detection and isolation of cases^[^2,5^]^. Although surveillance systems have been strengthened in Bangladesh and India, they continue to struggle with the timely detection of outbreaks and environmental reservoir monitoring^[^2,3^]^.

Besides scientific advancement, regulatory readiness will determine how quickly Nipah vaccine deployment progresses. Ordinary large-scale efficacy trials may not be achievable because outbreaks occur sporadically and unpredictably. Emergency use authorizations, adaptive trial designs, and immunobridging should be prioritized in regulatory strategies. Many international initiatives, including the WHO R&D Blueprint and CEPI’s 100 Days Mission, call for expedited regulatory pathways to enable rapid vaccine development and distribution during times when new infectious diseases arise^[^10,11^]^. Coordinating national regulatory authorities with international health organizations is vital to providing timely access as soon as vaccine candidates are found to be safe and effective.

Equitable distribution of future Nipah vaccines, once available, is a critical consideration. The COVID-19 pandemic exposed significant differences in global vaccine supply, especially among low- and middle-income countries that have the highest risk of zoonotic disease spillovers. To avoid these same inequities in future pandemics, proactive strategies (such as regional production, technology transfer, and advance purchase mechanisms) must be implemented. It will also be necessary to improve the capacity to produce vaccines in endemic countries, with the help of both international partnerships and funding organizations, so as to provide vaccines in a timely and cost-effective manner^[^12,13^]^.

In light of the delayed responses, urgent action is needed. Accelerated vaccine development and clinical trials are crucial, with platforms that have already demonstrated their usefulness during COVID-19 (e.g., mRNA or viral vectors) being given priority^[^7^]^. Strengthening international collaborative research, increasing funding for therapeutics development, and enhancing molecular surveillance can contribute to filling critical gaps^[^5,7^]^. Public health interventions, such as community education, safe treatment of food products (e.g., date palm sap), and bat population surveillance, are critical to avoiding spillover^[^2,3^]^. The absence of appropriate interventions will lead to a significant and ongoing recurrent threat to regional and global health security^[^1–3^]^.

The Nipah viral disease remains an under-recognized global health threat that can lead to disastrous outcomes. Targeted and coordinated efforts to vaccinate against Nipah will need to take place if the described threats are to be addressed effectively through the use of successful vaccines. As part of this coordinated global vaccination effort, it is important to conduct clinical studies quickly, strengthen regulations for the approval of vaccines for Nipah, enhance surveillance systems, and deliver vaccines equitably to those requiring vaccinations. Without global commitment to these efforts, the Nipah viral disease will remain a recurring threat to health security at both regional and global levels.

## Data Availability

No data were generated or analyzed for this study.

## References

[1] World Health Organization. Nipah virus. Geneva: World Health Organization; 2026 [Accessed 10 April 2026]. https://www.who.int/news-room/fact-sheets/detail/nipah-virus

[2] World Health Organization. Nipah virus infection – Bangladesh. Geneva: World Health Organization; 2025 [Accessed 10 April 2026]. https://www.who.int/emergencies/disease-outbreak-news/item/2025-DON582

[3] World Health Organization. Nipah virus infection – India. Geneva: World Health Organization; 2026 [Accessed 10 April 2026]. https://www.who.int/emergencies/disease-outbreak-news/item/2026-DON593

[4] Aditi SM Shariff M. Nipah virus infection: a review. Epidemiol Infect 2019;147:e95. 30869046 10.1017/S0950268819000086 PMC6518547

[5] Kiran V Girigoswami K Girigoswami A. Nipah virus: transmission dynamics of a zoonotic outbreak and therapeutic challenges. Biomed Res Ther 2025;12:7350–71.

[6] Singh RK Dhama K Chakraborty S. Nipah virus: epidemiology, pathology, immunobiology and advances in diagnosis, vaccine designing and control strategies—a comprehensive review. Vet Q 2019;39:26–55. 31006350 10.1080/01652176.2019.1580827 PMC6830995

[7] Madhukalya R Yadav U Parray HA. Nipah virus: pathogenesis, genome, diagnosis, and treatment. Appl Microbiol Biotechnol 2025;109:158. 40593310 10.1007/s00253-025-13474-6 PMC12214056

[8] Coalition for Epidemic Preparedness Innovations. Establishing the world’s largest Nipah virus vaccine reserve. 2024 [Accessed 10 April 2026]. https://cepi.net/establishing-worlds-largest-nipah-virus-vaccine-reserve

[9] University of Oxford. World’s first Phase II Nipah virus vaccine trial launch. 2025 [Accessed 2026 Apr 10]. https://www.ox.ac.uk/news/2025-12-15-worlds-first-phase-ii-nipah-virus-vaccine-trial-launch

[10] World Health Organization. WHO R&D blueprint for epidemics. Geneva: World Health Organization [Accessed 10 April 2026]. https://www.who.int/teams/blueprint/who-r-and-d-blueprint-for-epidemics

[11] Coalition for Epidemic Preparedness Innovations (CEPI). 100 days mission. Oslo: CEPI [Accessed 10 April 2026]. https://cepi.net/100-days-mission

[12] Gavi TVA. Vaccine Alliance 2023. Gavi; Accessed 10 April 2026. https://www.gavi.org/vaccineswork/vaccine-alliance-2023

[13] Srinivasan A Gopinath KG. Global emergence of Nipah virus: epidemiology, pathogenesis, diagnosis and treatment strategies. Front Microbiol 2021;12:8494620.

